# Frailty and sociodemographic and health factors, and social support network in the brazilian elderly: A longitudinal study

**DOI:** 10.1590/1980-220X-REEUSP-2021-0192

**Published:** 2021-12-08

**Authors:** Jack Roberto Silva Fhon, Luípa Michele Silva Cabral, Suellen Borelli Lima Giacomini, Nayara Araújo dos Reis, Marcela Cristina Resende, Rosalina Aparecida Partezani Rodrigues

**Affiliations:** 1 Universidade de São Paulo, Escola de Enfermagem, Departamento Médico-Cirúrgico, São Paulo, SP, Brazil.; 2 Universidade Federal de Goiás, Unidade Acadêmica de Biotecnologia, Departamento de Enfermagem, Catalão, GO, Brazil.; 3 Universidade de São Paulo, Escola de Enfermagem de Ribeirão Preto, Departamento Enfermagem Geral e Especializada, Ribeirão Preto, SP, Brazil.

**Keywords:** Frail Elderly, Aged, Geriatric Nursing, Longitudinal Studies, Risk Factors, Anciano Frágil, Anciano, Enfermería Geriátrica, Estudios Longitudinales, Factores de Riesgo, Idoso Fragilizado, Idoso, Enfermagem Geriátrica, Estudos Longitudinais, Fatores de Risco

## Abstract

**Objective:**

To identify and analyze the sociodemographic and health factors and the social support network of the elderly associated with frailty in the assessments carried out between 2007/2008 and 2018.

**Methods:**

This is a longitudinal study with elderly people aged ≥65 years living in the community. The instruments used were those for Demographic Profile, the Mini Mental State Examination, the Functional Independence Measure, Lawton and Brody Scale, Geriatric Depression Scale, Minimum Relationship Map for the Elderly, and Edmonton Frail Scale. Descriptive analysis and linear regression were used, all tests with p < 0.05.

**Results:**

Of the 189 elderly in the study period (2007/2008–2018), most were 80 years old and over, with an average of 82.31 years old; they were women, with no partner, who lived with other family members and were retired. In the final analysis, regardless of age and sex, a decrease in functional independence, an increase in depressive symptoms, an increase in the number of self-reported illnesses, and an increase in the frailty score were observed.

**Conclusion:**

The factors that were associated with the increase in frailty of the elderly during the study period were age, female sex, and no partner. The health team, which includes nurses, shall be aware of changes and develop care plans to prevent or avoid their progression.

## RESUMO

**Objetivo:**

Identificar e analisar os fatores sociodemográficos, de saúde e rede de apoio social do idoso associados à fragilidade nas avaliações realizadas entre 2007/2008 e 2018.

**Método:**

Estudo longitudinal com idosos de idade ≥65 anos que vivem na comunidade. Foram utilizados os instrumentos de Perfil demográfico, Mini Exame do Estado Mental, Medida de Independência Funcional, Escala de Lawton e Brody, Escala de Depressão Geriátrica, Mapa Mínimo de Relações do Idoso e Escala de Fragilidade de Edmonton. Utilizou-se a análise descritiva e regressão linear, todos os testes com p < 0,05.

**Resultados:**

Dos 189 idosos no período do estudo (2007/2008–2018), a maioria tinha 80 anos e mais, com média de 82,31 anos; sexo feminino, sem companheiro, moravam com outros familiares e eram aposentados. Na análise final, independentemente da idade e do sexo, verificou-se diminuição da independência funcional, aumento dos sintomas depressivos, do número de doenças autorreferidas e aumento no escore da fragilidade.

**Conclusão:**

Os fatores que se associaram ao aumento da fragilidade do idoso no tempo de estudo foram idade, sexo feminino e sem companheiro. A equipe de saúde, que inclui a enfermagem, deve estar atenta às mudanças e elaborar planos de cuidados para prevenir ou evitar a sua progressão.

## RESUMEN

**Objetivo:**

Identificar y analizar los factores sociodemográficos, de salud y red de apoyo social del adulto mayor asociado a la fragilidad en las evaluaciones realizadas entre 2007/2008 y 2018.

**Método:**

Estudio longitudinal con adultos mayores con ≥65 años que viven en la comunidad. Se utilizaron los instrumentos de Perfil demográfico, Mini Examen del Estado Mental, Medida de Independencia Funcional, Escala de Lawton y Brody, Escala de Depresión Geriátrica, Mapa Mínimo de Relaciones del Anciano e Escala de Fragilidad de Edmonton. Se utilizó el análisis descriptivo y regresión linear, todos los tests con p < 0,05.

**Resultados:**

Entre los 189 adultos mayores en el período de la investigación (2007/2008–2018), la mayoría tenía 80 años o más, con edad promedia de 82,31 años; sexo femenino, sin pareja, vivían con otros familiares y eran jubilados. En el análisis final sin considerar la edad y el sexo, se averiguó la disminución de la independencia funcional, aumento de los síntomas depresivos, del número de enfermedades auto referidas y aumento en el score de fragilidad.

**Conclusión:**

Los factores que se asociaron al aumento de la fragilidad del adulto mayor en el tiempo de investigación fueron la edad, el sexo femenino y sin pareja. El equipo de salud, que incluye a enfermería, debe tener atención a los cambios y elaborar planes de cuidados para prevenir o evitar su progresión.

## INTRODUCTION

The world population is aging rapidly. Statistics for Brazil report an increase in the elderly people in recent years. The projection of elderly for 2050 is of 29.3% of the general population^(1)^.

Aging and frailty are closely connected. Many of the mechanisms involved in the development of the frailty syndrome are similar to those presented in aging, where there is no single cause, reflecting the accumulated damage in different systems, such as inflammation, loss of stem cell regeneration, DNA damage, decline in metabolism, hormone dysregulation, epigenetic factors, and loss of proteostasis^(2)^. There is no single definition of frailty, but it is indicated as a state of vulnerability with poor homeostasis resolution after a stressful event, which increases the risk of adverse health outcomes^(3–4)^. Furthermore, it can be determined or modified by biological and psychosocial factors, with the complex etiology of a multifactorial nature being emphasized, and it is related to the life trajectory^(5)^.

Multiple factors, such as psychosocial and socioeconomic status, insufficient social support, inadequate nutrition and physical activity, multi-morbidity and chronic diseases, are associated with frailty^(6)^, making the elderly vulnerable and susceptible to different adverse outcomes, such as falls, functional disability, hospitalization, institutionalization, and death^(7)^.

Considering that the biomedical perspective is predominant in the literature, definitions of frailty refer to the existence of a very complex interaction among physical, psychological, social, and environmental factors that need to be studied^(8)^. Furthermore, these factors are often ignored in the health context, and may represent risk factors for the development of this syndrome^(9)^.

Thus, the aging process shall be considered a result of biological factors, as well as of the social, economic, political, and demographic ones, in addition to the process of redefining the family as a social institution. The present study addresses this relevant topic and makes efforts to detect frailty in the home settings in a follow-up study, considering that in Brazil there are few follow-up studies for this population. Thus, it is urgent to identify situations that can be considered a risk to the life of the elderly, to support public policies directed to the more vulnerable elderly population. Therefore, the objectives of the study were to identify and analyze the sociodemographic and health factors, and the social support network of the elderly associated with frailty in the assessments carried out between 2007/2008 and 2018.

## METHOD

### Design of Study

This is an observational retrospective cohort study in which information was previously collected and it was possible to identify the main outcome of the study, in this case the frailty syndrome; the study was guided by the recommendations of the Strengthening the Reporting of Observational Studies in Epidemiology (STROBE). The study lasted 10 years, Time 1 (T1) was carried out between July 2007 and February 2008^(10)^, and Time 2 (T2) between December 2017 and July 2018. The research was carried out in an inland city of the state of São Paulo, Ribeirão Preto, Brazil.

### Population

The sampling process was probabilistic through two-stage cluster. In the first stage, the census sector was considered as the Sampling Primary Unit (SPU), with 30 census sectors being drawn, with probability proportional to the size of the number of households, from the 600 sectors in Ribeirão Preto. In the second, streets and blocks were drawn and 110 households in each sector were visited.

Confidence intervals for prevalence, estimated in post-strata defined according to sex and age, should consider 10% as the maximum threshold for error tolerance. As a form of prevention in case of refusals or non-responses, 993 elderly people were drawn, a number that results from corrections for the expected response rate of 80%, being the final number of the sample.

At T1, 515 elderly people were evaluated and at T2, 189. The inclusion criteria were being 65 years of age or older, both sexes, being able to verbally answer the questions asked by the interviewer without help, and living in the urban area of the city of Ribeirão Preto. The loss of 336 elderly people was due to 212 deaths and 114 that were not located.

### Data Collection

For the evaluation at both times (T1 and T2) the following instruments were used:

Demographic profile: to identify data such as sex (male and female), age (in years), marital status (single, married, widowed and divorced), level of education (in years), number of children, who the elderly person lives with (alone, with spouse and other family members), retirement (yes and no), and elderly income (reais). The number of self-reported diseases that the elderly person has and arrangements (alone, with spouse and other family members) were also identified.

Mini Mental State Examination (MMSE): test used to assess cognitive function. The instrument was translated and validated for the Portuguese language^(11)^ and the scale ranges from zero to 30 points with a cohort point of 20 points for illiterates, 24 for those with 1 to 4 years, 26.5 points for 5 to 8 years of education, 28 points for those between 9 and 11 years and 29 points for those with more than 11 years of education^(11)^.

Functional Independence Measure (FIM): This instrument was developed by the American Academy of Physical Medicine and Rehabilitation and the American Congress of Rehabilitation Medicine and reproduced and validated for Brazilian Portuguese^(12)^. It assesses performance for 18 joint tasks related to scales of self-care, transfers, locomotion, sphincter control, communication and social cognition that include memory, social interaction, and problem solving^(12)^. The FIM can be classified into: Total FIM, motor FIM, and cognitive FIM, consisting of activities that receive a score ranging from 1 (total dependence) to 7 (complete independence); so the total score ranges from 18 to 126 points^(12)^.

Lawton and Brody scale: It includes more complex social activities, evaluating the individual’s ability to live in the community and is validated for Portuguese^(13)^. Its score ranges from seven (highest level of dependence) to 21 (complete independence) and the elderly can be categorized as total dependence (7 points); partial dependence (8 – 20), and independence (21 points) that are those able to perform all instrumental Activities of Daily Living (IADL) without help^(13)^.

Edmonton Frail Scale (EFS): Scale of the group Canadian Initiative on Frailty and Aging (CIF-A)^(14)^, validated and reproduced for the Portuguese language^(10,15)^. This scale assesses nine domains represented by 11 items: cognitive area with the clock drawing test; general health status; functional independence; emotional support; use of medications; nutrition; humor; continence; functional performance at Timed up and go test for balance and mobility. The scale has a score from zero to 17 points, with the highest score representing a higher level of frailty^(10,15)^.

Geriatric Depression Scale (GDS): original scale that was created from the items that most strongly correlated with the clinical diagnosis of depression. The scale was translated and validated to Portuguese^(16)^, consisting of 15 dichotomous questions and having a cutoff point ≥ five as indicative of cases of probable presence of depressive symptoms.

Minimum Relationship Map for the Elderly (MMRI): with the purpose of identifying and characterizing the social support network for the elderly. It is a graphic instrument that is easy and quick to apply, which allows the identification and visualization of significant links. Consisting of four quadrants representing family, friends, community and relationships with social or health services^(17)^.

The size of the social support network corresponds to the number of records in the MMRI, according to the perception of the elderly. For the present study, the quantitative number of contacts that the elderly person has in the respective quadrants was used.

### Data Analysis and Treatment

The collected data were entered into the software *Microsoft Excel* ^®^ by means of double typing and then a consistency analysis to compare them was performed; and later they were exported to the statistical software Statistical Package for the Social Sciences – SPSS v. 25.0.

In the descriptive phase, measures of central tendency and dispersion were used, and tests were carried out to compare means (T tests) in numerical variables or proportions (Chi-square) in categorical variables for the two moments of the study (T1 and T2). Furthermore, differences between scores were identified (T2 – T1).

In the analytical phase, the multiple linear regression technique was used to identify associations between sociodemographic variables (sex [male and female], age and marital status [with and without a partner]), health (cognitive status [MMSE], functional independence [FIM], Instrumental Activities of Daily Living [IADL], Depressive symptoms [GDS], and number of morbidities) and social support (number of family members, friends, community and health services [MMRI]) with frailty (Edmonton Scale). At this stage, we chose to present the longitudinal assessment (differences between T2 and T1) and the cross-sectional assessment (only T2).

Differences between the scores (T2 – T1) of the variables related to the Scales were calculated and subsequently classified into three categories, according to the cutoff points equivalent to the respective tertiles of the distributions. The list of morbidities was self-reported. All variables with more than two categories were treated as indicator (*dummy*) variables and the significance level adopted for all tests was α = 5%.

### Ethical Aspects

The study was approved by the Research Ethics Committee of the Nursing School of Ribeirão Preto of Universidade de São Paulo, complying with the requirements of CNS Resolution 466/2012 with protocol no. 0825/2007 and CAAE no. 68429117.7.0000.5393. Study participants signed the Free Informed Consent Form (FICF) in duplicate, one being delivered to the participant and the other remaining with the researcher.

## RESULTS

In a 10-year follow-up, it was found that out of the 515 elderly in T1, 212 died and there were 114 elderly who were not found, as they changed address (32), were not found in up to three visits (35), refused to participate (40), or were hospitalized (7) during the period of information collection.

However, of the 189 survivors identified at T2, most were 80 years old or older (64.0%), with a mean of 82.52 (5.49); were women (70.4%); with no partner (63.0%); lived with family members (56.5%), with an average of 2.98 (0.52) people in the elderly’s household, and were retired (63.5%) (Table 1).

**Table 1.** Demographic profile of the elderly living at home in 2017/2018. Ribeirão Preto, SP, Brazil, 2021.

Variables	Categories	Mean (±SD)	95% CI	n	%
Sex	Female			133	70.4
	Male			56	29.6
Age (years)	75–79	82.31 (5.49)	81.52–83.10	68	36.0
	80 or more			121	64.0
Level of education (years)		4.99 (4.71)	4.31–5.67		
Marital status	With no partner			119	63.0
	With partner			70	37.0
Live children		3.19 (2.25)	2.87–3.51		
With whom the elderly lives	Alone	2.98 (0.52)	1.96–4.01	44	23.3
	Spouse			40	21.2
	Other family members			105	56.5
Retired	Yes			120	63.5
	No			69	36.5
Income (Reais)		1,047.13 (1,573.14)	821.40–1,272.86		

SD = Standard deviation; CI = Confidence Interval.

As for the clinical evaluation, it was found that over the time (T2) of the research there was an increase in cognitive deficit (p < 0.001), presence of depressive symptoms, and mean frailty score (p < 0.001). Moreover, a decrease was observed in the scores mean for functional independence (p < 0.001), Instrumental Activities of Daily Living (IADL) (p = 0.01), and number of self-reported diseases. As for social support, it was found that there was a decrease in the support from the family (p < 0.001), community, friends, (p < 0.001) and health service (p = 0.02) (Table 2).

**Table 2.** Clinical characteristics of elderly people living at home in 2007/2008 and 2017/2018. Ribeirão Preto, SP, Brazil, 2021.

Variables	T1			T2			p*
	N	%	Mean (SD)	N	%	Mean (SD)	
**Cognitive status**							
No deficit	129	68.3		105	55.6		<0.001
With deficit	60	31.7		84	44.4		
**Frailty**			3.64 (2.44)			5.75 (2.96)	<0.001
Functional independence measure			121.69 (6.44)			116.33 (18.82)	<0.001
Instrumental activities			18.52 (3.63)			17.41 (4.10)	0.01
**Geriatric depression scale**			3.32 (2.83)			3.98 (3.03)	0.001
No symptoms	142	75.1		119	63.0		0.87
With symptoms	47	24.9		67	35.4		
**Number of diseases**			5.57 (3.38)			5.49 (3.44)	0.766
**Social support**							
Family			3.39 (2.04)			2.68 (1.89)	<0.001
Community			0.01 (0.07)			0.04 (0.28)	0.13
Friends			0.85 (1.45)			0.13 (0.51)	<0.001
**Health services**			0.03 (0.16)			0.00 (0.00)	0.02

* p-value for comparison tests.

In the final analysis with the model adjustment for the participants’ age and sex, it was observed that over time there is a decrease in functional independence, an increase in depressive symptoms and in the number of self-reported illnesses, with greater probability of increase in the frailty score. In the cross-sectional analysis, an association was identified between functional independence and depressive symptoms with an increase in the score on the frailty syndrome scale (Table 3). However, there was no association with social support in the longitudinal and cross-sectional analyses.

**Table 3.** Factors associated with frailty in a longitudinal and cross-sectional assessment of elderly people living at home. Ribeirão Preto, SP, Brazil, 2021.

Variables	Longitudinal assessment (Frailty Time 2 – Time 1)		Cross-sectional assessment (Frailty Time 2)	
	Beta	95% CI	Beta	95% CI
**Sex (female)**	0.161	–0.611 – 0.934	0.808	0.019 – 1.596
**Age (years)**	–0.009	–0.077 – 0.059	0.021	–0.049 – 0.091
**Functional independence**				
1st third	1.509	0.585 – 2.433	3.346	2.419 – 4.272
2nd third	0.092	–0.773 – 0.956	0.873	–0.013 – 1.758
3rd third	0		0	
**Depressive symptoms**				
1st third	0		0	
2nd third	1.022	0.105 – 1.940	0.536	–0.399 – 1.471
3rd third	1.608	0.668 – 2.548	1.632	0.671 – 2.592
**Self-reported diseases**				
1st third	0		–	
2nd third	0.365	–0.525 – 1.255	–	–
3rd third	1.443	0.555 – 2.331	–	–

## DISCUSSION

It was identified that, over 10 years, the decrease in functional independence, increase in depressive symptoms and morbidities and, in the cross-sectional analysis, decrease in functional capacity and increase in depressive symptoms were related to frailty. On the other hand, the relationship with the social support network was not identified.

The demographic data presented in this study are similar to international and national studies^(18–19)^. Demographic and epidemiological changes have brought an absolute and relative increase in the number of elderly people in the world, from the richest to the poorest regions. At the same time, these changes, in most cases, are followed by chronic diseases, syndromes that interact with social and economic problems^(20)^.

Frail people are more likely to have unfavorable outcomes after events arising from chronic diseases, medical interventions, and interventions that bring no benefit or that may even harm the health of the elderly. In addition, the higher health care costs of frail elderly may simply reflect their greater health care needs for health care services and the family itself^(20)^.

In a follow-up study carried out in Germany with 2,217 elderly people, the authors identified that those categorized as frail went from 6.2% to 12% and the pre-frail from 58.3% to 56.2%, and non-frail from 35.5% to 31.8%. It was identified that health care costs increased in frail elderly, especially in hospitalization and informal nursing care. Furthermore, among the frailty domains, the onset of exhaustion was associated with an increase in total health care costs^(21)^.

This same condition can be observed in the study carried out in Australia, in which 415,769 people aged 65 years and over were considered frail and 1.7 million, pre-frail. Projections carried out for 2027 indicate that the number of elderly people considered frail will increase to 609,306 and the pre-frail to 2,248,977^(22)^, which can lead to increased hospitalization costs, limitations, and decreased functional independence.

This decrease in independence was identified in the follow-up results and in the cross-sectional analysis associated with the frailty syndrome. The aging process can lead to a decline in physical fitness and functional capacity, which can worsen with a sedentary lifestyle, making the elderly dependent on care^(23)^, developing dynapenia, which is the clinical manifestation of frailty, evidenced in muscle wasting and decrease of muscle strength^(7)^.

The study showed that there was an association between frailty and decreased functional independence in the elderly. These data were identified in the Italian study that shows the assessment of 366 elderly people, with Edmonton Frail Scale and that also identified an association with basic activities (B = –0.512, p < 0.001) and instruments of daily life (B = –0.338, p < 0.01)^(24)^.

Functional capacity encompasses the health-related attributes that allow a person to be and do what is important to them and consists of the intrinsic capacity, the characteristics of the environment that affect this capacity, and the interactions between the person and these characteristics^(20)^ and, as it decreases, more attention shall be paid to preserving it.

Physical activity is considered a useful tool for successful aging, as well as for the prevention of the adverse effects of old age, since sedentary lifestyle increases the risk of mortality, chronic diseases, and functional and cognitive impairment^(25)^.

As for the presence of depressive symptoms during the follow-up, it was associated with frailty. Depression is a disease with somatic and psychological symptoms that can recur throughout a person’s life, with a considerable impact on the quality of life of the elderly^(19)^ and, like frailty, is a common medical condition in the elderly. In a systematic review, the authors found a reciprocal interaction between depression and frailty, and that each condition is associated with an increased prevalence and incidence of the other, being considered a risk factor for the development of both conditions^(2)^.

In a study carried out in Europe with 58,152 participants over 50 years of age to assess the interdependencies between frailty and depression over time, the authors identified that both variables reciprocally influence each other over time, but indicate that frailty and depression can be affected by common causes, both in the short and long term^(26)^.

It was also found that the greater number of diseases leads to an increase in frailty in the elderly. Brazilian researchers have observed that diseases such as Diabetes Mellitus, heart disease, and osteoarticular disease that are found in the elderly were associated with frailty^(27)^. With frailty, chronic diseases often coexist, increasing the risk of adverse health events, in addition to being strongly associated with decreased mobility, increased risk of falls, polypharmacy, decreased cognitive function, malnutrition, hospitalization, increased cost of health care and mortality^(7)^.

With the increase in the number of diseases in the elderly, there is a probability that this population uses more than five medications a day. Although these contribute to improving the quality of life, they can have a negative impact and an increase in adverse events^(28)^.

In this study, no association was identified between the social support network and frailty. However, there was a decrease in the social support network over time. A Korean study showed that social support is essential for the elderly with this syndrome. The researchers found that the frequency of contact with friends was the most significant among the elderly. In addition, those who have friends visiting them monthly or rarely were more likely to be frail compared to the group who had daily contact^(18)^.

In the United Kingdom, in an evaluation with 1,094 elderly people, a similar result was identified. Family support was not associated with frailty syndrome, although it improves the prediction model for frail hospitalized elderly with coronary heart disease^(29)^.

Social relationships are vital for the psychological and physical health of the elderly and an inadequate network leads to feelings of isolation and loneliness among them^(30)^. Social support includes real or perceived resources provided by third parties that allow the elderly to feel cared for, appreciated, and part of a network, promotes health and increases well-being and quality of life, providing positive experiences, socially active roles, or rather, helping to deal with stressful events^(29)^.

The study had limitations: 1) During the process, there were losses due to death, change of residence of the elderly, and difficulty in locating the study participant and 2) difficulty in locating the elderly, since the municipality is a reference in the health area and health services end up assisting the residents’ relatives, who live in other municipalities.

The longitudinal study provided researchers with opportunities for follow-up in the assessment of frailty, since this syndrome increases the risk of several health problems, leading the elderly to dependence, institutionalization, and death. In addition, it will allow support to health professionals, especially nurses, to plan individualized care to prevent this syndrome. The elderly assessment regarding the frailty syndrome aims to plan care in the primary care service (home and in the Health Unit), providing better living conditions.

## CONCLUSION

The importance of this study was the follow-up of the elderly who live at home. Most of the elderly were women, with no partner and a mean age of 82.31 years, considered high for the Brazilian population. The frailty syndrome, in this population, is related to the decrease in functional independence, the presence of depressive symptoms and the increase in the number of chronic diseases over ten years.

Although the social support network was not shown to be associated in this study, the interpersonal relationship through social support is a variable that should be considered in the elderly’s health. These data are relevant, as they offer health professionals, especially nurses, more concrete data for the development of individualized care plans directed to elderly people in a pre-frail or frail stage, with a low social support network, as well as for the implementation of public policies for interaction groups among the elderly.

## Financial support

This work was carried out with the support of the Coordenação de Aperfeiçoamento de Pessoal de Nível Superior – Brazil (CAPES) – Financing Code – 001.

The study was subsidized by the Conselho Nacional de Desenvolvimento Científico e Tecnológico-CNPq. Process number 305565/2016-8.
